# How do surgically treated multiligamentous knee injuries affect overall complication rate and especially stiffness? A systematic review

**DOI:** 10.1186/s43019-025-00270-9

**Published:** 2025-05-01

**Authors:** Lucas Martorell de Fortuny, Alexandre Santoli, Vasileios Giovanoulis, Angelo V. Vasiliadis, Simone Perelli, Joan Carles Monllau, Az-Eddine Djebara, Nicolas Pujol

**Affiliations:** 1 Department of Orthopaedic, Centre Hospitalier de Versailles, 78150 Le Chesnay, France; 2 ICATKnee, Institut Català de Traumatologia I Medicina de L’Esport (ICATME), Hospital Universitari Dexeus, Universitat Autònoma Barcelona, 08028 Barcelona, Spain

**Keywords:** Knee, Multiligamentous, Complications, Stiffness

## Abstract

**Background:**

Multiligamentous knee injuries (MLKIs), defined as injuries involving at least two of the four primary knee ligaments, are rare but severe, with potentially limb- or life-threatening complications. Despite numerous publications, the low incidence and heterogeneity of injury patterns limit high-level evidence for optimal surgical timing, technique, and management of complications. This systematic review aims to consolidate the available evidence on MLKI surgery complications, with a particular focus on arthrofibrosis as the underlying cause of stiffness, infection, and graft failure.

**Methods:**

This systematic review was conducted following Preferred Reporting Items for Systematic Reviews and Meta-analyses (PRISMA) 2020 guidelines and registered in the International Prospective Register of Systematic Reviews (PROSPERO) (no. CRD42024618025). A comprehensive search of PubMed, EMBASE, and MEDLINE from January 2013 to November 2024 identified studies reporting complications in surgically treated MLKIs with at least a 12-month follow-up. The studies were screened independently by two reviewers. Data on demographics, injury mechanisms, surgical techniques, and complication outcomes were extracted. Study quality was assessed using the Methodological Index for Non-Randomized Studies (MINORS).

**Results:**

A total of 33 studies with 2863 patients met the inclusion criteria. The mean age was 32.4 years (standard deviation, SD ± 5.37), with males constituting 69.4% of the sample. Arthrofibrosis was the most common complication, requiring surgical management in 8.4% of cases. Graft failure was reported in 5%, while infection, the third most common complication, occurred in 2.86% of cases. Management of lack of range of motion varied, with manipulation under anesthesia and arthroscopic arthrolysis utilized. Surgical timing also influenced outcomes; 54.2% of patients underwent acute surgery (< 21 days), which seems to be associated with increased stiffness rates.

**Conclusions:**

This systematic review highlights the complexity of managing MLKIs, with a 19.2% overall complication rate. Stiffness demanding reoperation remains a rare but a significant challenge, underscoring the need for standardized treatment protocols. However, the included studies demonstrate heterogeneity and lack high methodological rigor, highlighting the need to account for these limitations.

## Introduction

Multiligamentous knee injuries (MLKIs) are defined as the injury of at least two of the four main ligaments of this joint. Schenk classification [1] is based on the anatomical pattern of ligament injury and is widely used in literature, while the French Society of Orthopedic Surgery and Traumatology (SOFCOT) classification [2] is focused on the mechanism of injury, providing insights into the trauma’s dynamics and helping in planning surgical or conservative treatment. Although it is a rare entity [3–8], with an incidence of 0.02–0.2% of all injuries treated in orthopedic surgery, this figure likely underestimates the actual number of cases, as it does not account for self-reduced knee dislocations and mis- or under-diagnosed injuries [9, 10].

Despite the large number of publications on the subject, the low incidence together with the great heterogeneity of injury patterns limit the possibility to reach definitive conclusions on the basis of high-level scientific evidence regarding the optimal time to perform surgery, whether to perform it in one or two stages, whether to repair or reconstruct, and even on which is the most appropriate surgical technique and the most suitable graft. [4–7, 11].

There is a consensus on two key points regarding MLKIs. First, they are severe injuries associated with a high rate of complications and comorbidities, and in some cases, they can be limb- or even life-threatening. Second, nonoperative treatment is considered inferior to surgical management and should be reserved only for patients who are unsuitable for surgery, such as those who are frail or sedentary [3, 4, 7, 9, 11, 12]. The first point is consistently emphasized—often in similar terms—in the majority of publications discussing MLKIs [3–9, 11–30].

Despite the numerous publications describing the complications associated with the surgical treatment of MLKIs, the current evidence remains limited and insufficient to establish definitive conclusions.. Consequently, the apparent high degree of awareness among orthopedic surgeons regarding the severity and frequency of complications is primarily based on the findings of descriptive studies [8, 9].

The purpose of this study was to provide a systematic review of all available studies on surgically treated MLKIs that report complication outcomes. The goal was to determine the overall complication rate, with a particular focus on complications such as arthrofibrosis; the pathological process leading to stiffness, which may manifest as restricted range of motion (ROM) postoperatively; infection; and graft failure.

## Methods

### Search strategy and design

A systematic review of the available evidence was conducted in accordance with the Preferred Reporting Items for Systematic Reviews and Meta-analyses (PRISMA 2020) [13]. The review protocol has been registered in the International Prospective Register of Systematic Reviews (PROSPERO) with the registration number CRD42024618025. The following online databases were utilized for the search: PubMed, EMBASE, and MEDLINE. The search, conducted from January 2013 up to November 2024, imposed no date restrictions. The keywords used, along with their MeSH terms in all possible combinations, included: “knee dislocation,” “multiligament,” “multiligamentous,” “knee,” “injury,” and “complication.” A minimum mean follow-up of 1 year was required to ensure the assessment of all early complications and outcomes.

### Eligibility criteria

Studies published in English were included if they met the following criteria: (1) the sample exclusively consisted of patients with injuries to at least two of the four primary knee ligaments who were treated surgically, (2) the results explicitly reported complication outcomes, (3) it had a minimum follow-up at least 12 months, (4) sample size exceeded five patients, and (5) it was a full-text publication written in English. Conversely, studies were excluded if they met any of the following criteria: it (1) focused on revision surgeries for MLKIs, (2) exclusively targeted MLKI type knee dislocation (KD) 5, (3) employed treatment based solely on repair, and (4) included external fixation as a criterion for inclusion. Additionally, review articles, systematic reviews, meta-analyses, case reports, and opinion articles were excluded. 

### Selection of studies

The study selection process was carried out independently by two reviewers. Articles were initially screened on the basis of their titles and abstracts, with full-text articles obtained when further evaluation was required. After excluding studies that did not meet the criteria, the full texts of the remaining articles were thoroughly reviewed. Disagreements between the reviewers were resolved through discussion with a third author, who provided an independent assessment to ensure consensus and minimize bias in data extraction. Additionally, the references of the included articles were examined to identify other relevant studies for inclusion. Excluded studies and reasons were listed.

### Data extraction

Data extraction was conducted using a standardized form to ensure consistency across all eligible studies by the two same authors. The data collected included: (1) author, title, year of publication, journal, and study design; (2) sample information (sample size, gender, mean age and body mass index); (3) injury data (mechanism of injury, Schenck classification [1], and associated injuries); (4) treatment information (time to surgery, single or staged surgery, and number of isolated repair cases); (5) follow-up information and details on the number and types of complications, including preoperative and postoperative complications (e.g., arthrofibrosis, infection, and graft failure).

### Quality assessment of the eligible studies

The quality of all included studies was assessed using the Methodological Index for Non-Randomized Studies (MINORS) [14] criteria. Each item was rated on a scale from 0 to 2, with a maximum possible score of 16 for noncomparative studies and 24 for comparative studies. The scoring was independently performed by the same two authors.

## Results

### Literature review and general study characteristics

**Fig. 1:**
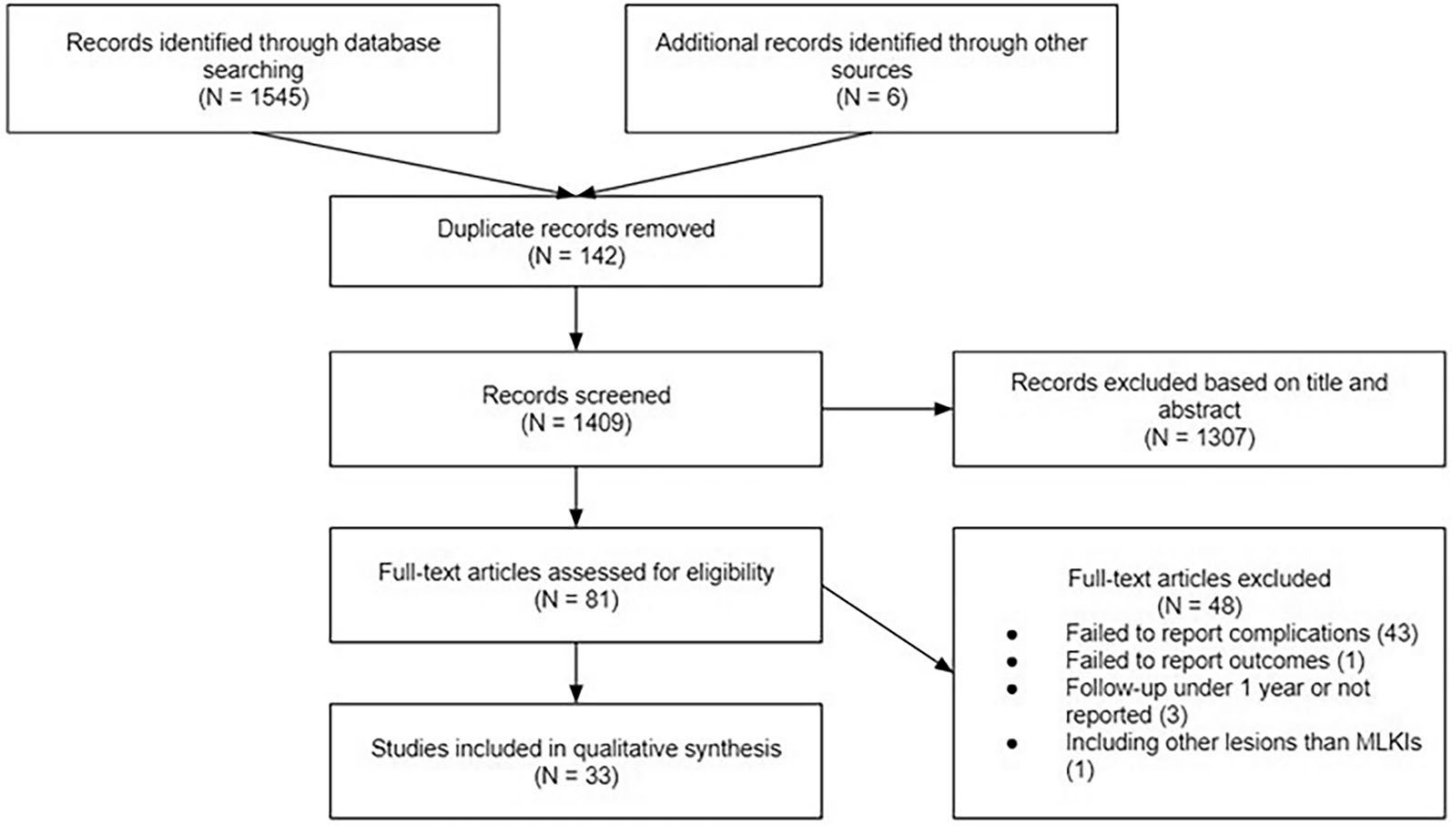
Flow diagram summarizing literature research and reasons for the excluded articles

The initial search identified 1545 studies through database searching, with an additional 6 studies found through other sources. After removing 142 duplicate records, 1409 studies remained for screening on the basis of their titles and abstracts. Of these, 1307 studies were excluded, leaving 81 full-text articles to be assessed for eligibility. Following this evaluation, 48 articles were excluded for the following reasons: 43 failed to report complications, 1 failed to report outcomes, 3 had a follow-up period under 1 year or did not report follow-up, and 1 included other lesions beyond MLKIs. Finally, 33 studies [15–47] encompassing a combined total of 2863 patients were included in the qualitative synthesis. A total of 26 [15–22, 24–29, 31, 33–38, 40–45] studies were retrospective, 6 were [21, 23, 30, 32, 39, 46] prospective, and 1 was a randomized control trial [47]. In total, 22 articles were case series, 10 were comparative studies, and 1 was a clinical trial. The result of our research is shown in the PRISMA flow diagram (Fig. 1), and the baseline characteristics of all studies included are reported in Table 1.

**Table 1:** Baseline characteristics of included studies

Author (journal, year)	Type of article	N	Schenk classification							Male (%)	Age (years)	Body mass index (BMI) (kg/m^2^)	High energy (%)	Follow-up	
			KD I (%)	KD II (%)	KD III (%)	KD II M (%)	KD III L (%)	KD IV (%)	KD V (%)					Min	Med
Ibrahim et al. [13]	Retrospective (RS)	20	0 (0)	0	–	0	20 (100)	0	0	20 (100)	26.4	–	11 (55)	24	44
Freychet et al. [33]	RS	40	6 (15)	0	–	0	20 (59)	12 (30)	2 (5)	36 (90)	24	–	23 (57.5)	24	63.6
Cook et al. [14]	RS	133	78 (58.7)	1 (0.8)	–	24 (18)	22 (16.5)	4 (3)	4 (3)	101 (75.9)	26	28.8	52 (39.1)	12	–
Wajsfisz et al. [15]	RS	53	0	53 (100)	–	0	0	0	0	46 (86.8)	29.8	–	19 (35.8)	12	49
Lee JH-Y et al. [16]	Prospective (PS)	42	16 (38.1)	1 (2.4)	–	5 (11.9)	10 (23.8)	7 (16.7)	3 (7.1)	32 (76.2)	28.9	33.2	25 (59.5)	12	14.2
Ridley et al. [34]	RS	126	–	–	–	–	–	–	–	96 (76.2)	25.8	28.8	76 (60.3)	12	24
Werner et al. [17]	RS	215	121 (56.3)	9 (4.2)	–	24 (11.2)	39 (18.1)	22 (10.2)	0	165 (76.7)	32	–	–	24	69.6
Zhang et al. [18]	RS	59	16 (27.1)	10 (16.9)	–	7 (11.9)	12 (20.3)	9 (15.3)	5 (8.5)	50 (84.7)	43.7	–	44 (74.6)	21	30
Tardy et al. [19]	RS	39	18 (46.2)	0	–	13 (33.3)	8 (20.5)	0	0	31 (79.5)	34.1	24.2	22 (56.4)	12	57
Hongwu et al. [35]	RS	13	0	0	–	0	12 (92.3)	1 (7.7)	0	8 (61.5)	37.8	–	6 (46.2)	24	32.6
Suh et al. [36]	RS	20	0	2 (10)	–	7 (35)	7 (35)	4 (20)	0	14 (70)	37	–	13 (65)	12	18.7
Barret et al. [37]	RS	32	15 (46.9)	2 (6.2)	–	12 (37.5)	0	3 (9.4)	0	21 (65.6)	30	–	19 (59.4)	28	87
Lau et al. [38]	RS	1350	871 (64.5)	88 (6.5)	391 (29)	–	–	0	0	887 (65.7)	40.1	–	–	12	–
Djebara et al. [20]	RS	29	12 (41.4)	0	–	0	16 (55.2)	1 (3.4)	0	26 (89.7)	30.2	26.5	16 (55.2)	69.6	90
Goyal et al. [21]	PS	27	0	8 (29.6)	–	11 (40.8)	6 (22.2)	2 (7.4)	0	24 (88.9)	33.4	26.7	27 (100)	24	24
Billiers et al. [22]	RS	20	6 (30)	0	–	5 (25)	7 (35)	2 (10)	0	15 (75)	28.3	25.2	8 (40)	23.3	29.4
Mahmood et al. [23]	PS	51	41 (80.3)	3 (5.9)	6 (11.8)	-	-	1 (2)	0	37 (60.8)	30.6	–	12 (23.6)	36.88	46
Hantes et al. [39]	PS	26	15 (57.7)	7 (26.9)	–	2 (7.7)	2 (7.7)	0	0	21 (80.8)	27.4	25.4	26 (100)	71.46	105.38
Chao et al. [24]	RS	12	0	0	0	–	–	12 (100)	0	9 (75)	40.3	24.6	–	24	24
Khan et al. [25]	RS	27	4 (14.8)	4 (14.8)	–	8 (29.6)	7 (25.9)	3 (11.1)	1 (3.7)	27 (100)	35.8	–	–	24	–
Lutz et al. [40]	RS	32	32 (100)	0	0	0	0	0	0	27 (84.4)	32	25.3	11 (34.4)	24	57.35
Jokela et al. [41]	RS	25	0	0	–	25 (100)	0	0	0	17 (68)	44	29	19 (76)	22	82
Laprade et al. [26]	RS	194	163 (84)	4 (2.1)	–	16 (8.2)	6 (3.1)	5 (2.6)	0	83 (42.8)	34.5	–	0	24	42
Yazdi et al. [42]	RS	11	8 (72.7)	0	–	3 (27.3)	0	0	0	10 (90.1)	32	–	–	12	19
Zhang et al. [43]	RS	21	21 (100)	0	–	0	0	0	0	–	39.6	–	–	26	40
Bonadio et al. [44]	RS	13	13 (100)	0	–	0	0	0	0	9 (69.2)	32	–	7 (53.8)	24	44.6
Richter et al. [27]	RS	8	0	0	–	8	0	0	0	6 (75)	28	–	4 (50)	24	144
Kitamura et al. [45]	RS	31	22 (71)	0	–	9 (10)	0	0	0	25 (80.6)	28.6	–	3 (9.7)	24	72
Helito et al. [46]	PS	9	9 (100)	0	–	0	0	0	0	8 (88.9)	29.9	–	2 (22.22)	24	27,3
Dong et al. [47]	RCT	64	64 (100)	0	–	0	0	0	0	37 (57.8)	36.6	–	–	24	34,2
Godin et al. [28]	RS	20	14 (70)	1 (5)	–	3 (15)	2 (10)	0	0	14 (70)	17.7	25.7	2 (10)	24	37.1
Woodmas et al. [29]	RS	62	24 (38.7)	0	–	14 (22.5)	10 (19.4)	10 (19.4)	4	42 (67.7)	33.5	30.7	31 (50)	24	74
Alentorn et al. [30]	PS	39	19 (48.8)	2 (5.1)	–	8 (20.5)	8 (20.5)	2 (5.1)	0	28 (71.8)	39.1	–	–	12	27

Comparative studies achieved scores ranging from 16 to 19, with a mean of 17.2 (± 1.1), indicating moderate methodological quality. Noncomparative studies scored between 7 and 14, with a mean of 10.41 (± 1.56), similarly reflecting moderate quality. The most frequently missed criteria were blinded evaluation of endpoints (missed in 31 out of 32 studies), a priori sample size calculation (missed in 29 out of 32 studies), prospective data collection (missed in 25 out of 32 studies), and the management of loss to follow-up (missed in 17 out of 32 studies).

The mean follow-up was 50.3 (± 29.27) months. The mean sample size was 86.76 patients (range 8–1350). Most patients were male (69.39%) with an average age across all studies of 32.4 years (± 5.75). The mean BMI from the available studies was 27.24 kg/m^2^ (± 2.57). A total of 27 studies described the mechanism of injury (1366 patients). From the available data of this sample, 45.5% of the injuries were caused by high-energy accidents, 31.6% by sports accidents, 15.9% by low-energy accidents, and 7% by other mechanisms.

### Demographics and surgical management

In terms of lesion classification, all but one study [33] used Schenck’s classification to categorize injuries, with a total of 2737 patients. The most frequent lesion type was KD I (58.7%), followed by KD III (29.8%), KD II (7.1%), KD IV (3.7%), and KD V (0.7%). Within the KD III subgroup, medial complex injuries accounted for 48.8%, while lateral complex injuries were slightly more common at 51.2%.

Regarding treatment strategies, data on surgical timing and type of surgery were available for 994 and 2116 patients, respectively. Among these, 45.8% underwent delayed reconstruction, while 54.2% underwent acute repair or reconstruction. Additionally, 83.8% of patients had single-stage surgeries, whereas 16.2% underwent staged procedures. Although not all studies reported on surgical techniques, data on the use of repair techniques were available for 1051 patients, of whom only 79 (7.5%) had associated repairs. These findings, along with associated lesions, are summarized in Table 2.

**Table 2:** Surgery characteristics of included studies

Author	Timing		Surgery stages		Technique		Associated injuries				
	Acute	Delayed (> 3 week)	Single stage	Staged	Extraarticular reparation	Only reconstruction	Vascular	Nerve	Extensor apparatus	Chondral	Meniscal
Ibrahim et al. [13]	20	0	20	0	0	20	9	18	0	13	22
Freychet et al. [33]	–	–	20	20	3 (3 Posterolateral Corner (PLC))	37	0	0	0	0	0
Cook et al. [14]	63	70	–	–	12	121	4	26	0	0	0
Wajsfisz et al. [15]	10	43	–	–	8	45	0	0	0	0	0
Lee JH-Y et al. [16]	20	22	–	–	0	42	2	7	0	8	18
Ridley et al. [34]	–	–	–	–	–	–	5	18	0	0	0
Werner et al. [17]	–	–	–	–	–	–	10	18	0	0	0
Zhang et al. [18]	48	11	–	–	–	–	0	0	0	0	0
Tardy et al. [19]	28	11	39	0	9 (9 PMC)	30	0	0	6	0	12
Hongwu et al. [35]	13	0	13	0	11 (10 MCL/1 MCL + LCL)	2	0	0	0	0	4
Suh et al. [36]	12	6	0	20	4 (4 MCL)	16	0	0	0	0	0
Barret et al. [37]		-–	–	–	0	32	0	0	0	0	15
Lau et al. [38]	–	–	1080	270	–	–	3	0	0	0	0
Djebara et al. [20]	16	13	–	–	0	29	0	8	0	0	10
Goyal et al. [21]	0	27	27	0	0	27	1	0	0	11	16
Billiers et al. [22]	13	7	20	0	0	20	2	0	2	5	6
Mahmood et al. [23]	21	30	51	0	0	51	0	1	0	0	0
Hantes et al. [39]	0	26	26	0	0	126	0	0	0	0	0
Chao et al. [24]	12	0	12	0	0	12	0	0	0	0	0
Khan et al. [25]	0	27	14	13	0	27	0	0	0	0	19
Lutz et al. [40]	–	–	32	0	0	32	0	0	0	0	9
Jokela et al. [41]	25	0	25	0	0	25	0	0	0	9	8
Laprade et al. [26]	153	41	194	0	0	194	0	0	0	59	107
Yazdi et al. [42]	–	–	–	–	0	11	0	0	0	0	0
Zhang et al. [43]	0	21	21	0	0	21	0	0	0	0	9
Bonadio et al. [44]	–	-–	13	0	0	13	0	0	0	0	0
Richter et al. [27]	2	6	–	–	0	8	0	0	0	0	0
Kitamura et al. [45]	0	31	31	0	0	31	0	0	0	0	0
Helito et al. [46]	–	–	9	0	0	9	0	0	0	0	0
Dong et al. [47]	64	0	64	0	32 (32 MCL)	32	0	0	0	5	17
Godin et al. [28]	9	11	20	0	0	20	0	0	0	7	11
Woodmas et al. [29]	10	52	42	20	–	–	5	5	0	0	0
Alentorn et al. [30]	–	–	–	–	0	39	0	2	0	17	30

### Complications

A total of 550 complications (19.2%) were recorded, including infections, arthrofibrosis underlying as cause of stiffness, venous thrombosis, hardware complication requiring removal, graft failure, compressive hematoma, nerve injury, amputation and death. All complications are presented in Table 3.

**Table 3:** Complications reported in the included studies

Author	Infection	Arthrofibrosis	Deep vein thrombosis	Hardware complication	Graft failure	Compressive hematoma	Postsurgical nerve	Amputation	Death	Total
	Total (%)	Total (%)	Total (%)	Total (%)	Total (%)	Total (%)	Total (%)	Total (%)	Total (%)	(%)
Ibrahim et al. [13]	0	4 (20)	0	0	0	1 (5)	0	0	0	5 (25)
Freychet et al. [33]	2 (5)	3 (7.5)	0	2 (5)	0	0	0	0	0	7 (17.5)
Cook et al. [14]	5 (3.8)	19 (14.3)	1 (0.7)	6 (4.5)	12 (9)	0	0	0	0	43 (32.3)
Wajsfisz et al. [15]	2 (3.8)	4 (7.6)	0	0	0	1 (1.9)	0	0	0	7 (13.2)
Lee JH-Y et al. [16]	3 (7.4)	3 (7.4)	0	3 (7.4)	3 (9.5)	0	0	0	0	12 (30.9)
Ridley et al. [34]	3 (2.4)	17 (13.5)	1 (0.8)	0	9 (7.4)	0	0	0	0	30 (23.8)
Werner et al. [17]	6 (2.8)	21 (9.8)	6 (2.8)	13 (6)	7 (3.3)	0	0	1 (0.5)	1 (0.5)	55 (25.6)
Zhang et al. [18]	3 (5)	2 (3.4)	0	0	5 (8.5)	0	0	0	0	10 (16.9)
Tardy et al. [19]	1 (2.6)	7 (18)	0	0	0	0	0	0	0	8 (20.5)
Hongwu et al. [35]	0	1 (7.7)	0	0	0	0	0	0	0	1 (7.7)
Suh et al. [36]	1 (5)	4 (20)	0	0	0	0	0	0	0	5 (25)
Barret et al. [37]	0	0	0	1 (3.1)	1 (3.1)	0	0	0	0	2 (6.2)
Lau et al. [38]	37 (2.7)	106 (7.9)	12 (0.9)	0	80 (5.9)	2 (0.2)	0	0	0	237 (17.6)
Djebara et al. [20]	1 (3.5)	3 (10.3)	0	2 (6.9)	0	0	0	0	0	6 (20.7)
Goyal et al. [21]	2 (7.4)	3 (11.1)	0	0	2 (7.4)	0	0	0	0	7 (25.9)
Billiers et al. [22]	0	1 (5)	0	5 (25)	0	0	1 (5)	0	0	7 (35)
Mahmood et al. [23]	2 (3.9)	0	0	2 (3.9)	0	0	0	0	0	4 (7.8)
Hantes et al. [39]	0	0	0	0	0	0	0	0	0	0
Chao et al. [24]	0	1 (8.3)	1 (8.3)	0	0	0	0	0	0	2 (26.7)
Khan et al. [25]	2 (7.4)	0	0	0	0	0	1 (3.7)	0	0	3 (11.1)
Lutz et al. [40]	1 (3.1)	0	0	0	1 (3.1)	0	0	0	0	2 (6.2)
Jokela et al. [41]	0	0	0	0	3	0	0	0	0	3 (12)
Laprade et al. [26]	1 (0.5)	18 (9.3)	3 (1.5)	5 (2.6)	9 (4.6)	0	0	0	0	36 (18,5)
Yazdi et al. [42]	0	0	0	0	0	0	0	0	0	0
Zhang et al. [43]	1 (4.8)	1 (4.8)	0	1 (4.8)	0	1 (4.8)	0	0	0	4 (19)
Bonadio et al. [44]	2 (15.4)	1 (7.7)	0	0	0	0	0	0	0	3 (23.1)
Richter et al. [27]	0	1 (12.5)	0	1 (12.5)	0	0	0	0	0	2 (25)
Kitamura et al. [45]	0	0	0	0	1 (3.2)	0	0	0	0	1 (3.2)
Helito et al. [46]	1 (11.1)	0	0	0	0	0	0	0	0	1 (11.1)
Dong et al. [47]	0	1 (3.1)	0	0	7 (21.9)	0	0	0	0	8 (12.5)
Godin et al. [28]	0	0	0	0	2 (10)	0	0	0	0	2 (10)
Woodmas et al. [29]	4 (6.5)	6 (9.7)	0	0	1 (1.6)	0	0	0	0	11 (17.7)
Alentorn et al. [30]	2 (5.2)	13 (33.3)	0	9 (23.1)	0	1 (2.6)	0	0	0	25 (64.1)
Total (%)	82 (14.9)	240 (43.5)	24 (4.4)	50 (9.1)	143 (26.2)	6 (1.1)	2 (0.4)	1 (0.2)	1 (0.2)	550

Arthrofibrosis emerged as the most common complication, with 240 cases requiring surgical management, representing an incidence of 8.4%. Notably, only ten case series [23, 25, 28, 37, 39–42, 45, 46] were entirely free of this complication. A standardized definition of stiffness was lacking across the studies as there is a lack of consensus on the diagnostic criteria [48]. Werner et al. [18] defined it as a loss of > 10° of flexion or extension. Similarly, there was no consensus on treatment indications, leading to variability in management approaches among the publications. Isolated mobilization under anesthesia (MUA) was performed in nine studies, arthroscopic arthrolysis was performed in six studies, and three studies differentiated between MUA and arthrolysis but did not clarify the indications. Additionally, one case of heterotopic ossification was treated with open arthrolysis, while 4 publications did not specify the treatment method. Only six studies, encompassing 19 cases of arthrofibrosis, reported the timing of surgical intervention, with an average time to surgery of 4.1 months. Of these cases, only two patients (0.8%) required reoperation.

The second most common complication was graft failure, with 143 reported cases (5% of the sample). In total, 14 of the included studies [16, 18, 21, 26, 28, 29, 32, 33, 37, 38, 40, 43, 45, 47] documented cases of graft failure; however, only two explicitly defined the term. Cook et al. [16] described graft failure as any case requiring reoperation owing to instability, or cases where radiology or physical examination indicated a failure of the previous reconstruction. Barrett et al. [37] defined it as reoperation for symptomatic instability or asymmetric gapping on stress X-ray greater than 2 mm. Of all the included studies, only six used objective methods to evaluate postoperative stability: stress X-rays were utilized in five studies, while KT-2000 arthrometers were used in two. Additionally, 30 studies [15, 17–32, 34–37, 39–47] employed functional tests to assess stability postoperatively, with the Lysholm test being the most common, followed by the International Knee Documentation Committee (IKDC) questionnaire.

Other complications were significantly less frequent. Infection was the third most common complication, with 82 reported cases (2.9%). Among these, treatment strategies were documented in only 24 cases: 15 were treated with antibiotics combined with surgical debridement, while 9 were managed with antibiotics alone. Hardware removal due to discomfort accounted for 50 cases (1.8%). In terms of vascular complications, 24 cases of deep vein thrombosis were reported (0.8%), along with 6 cases of compressive hematoma requiring surgical intervention (0.2%). Postsurgical nerve injuries were rare, with only two cases documented (0.07%), one of which eventually resolved. There were also two extreme complications: one case of amputation (0.04%) and one case of death (0.04%). Both cases, reported by Werner et al. [18], lacked specific details regarding the underlying causes of these complications.

#### Long-term complications

In total, two studies [18, 21] reported that four (0.2%) patients developed post-trauma arthritis and subsequently underwent total knee arthroplasty (TKA). Another study [39] found that 18 reconstructed knees (0.9%) exhibited radiographic signs of osteoarthritis. Additionally, a case with a 9.3-year follow-up was documented to exhibit minimal arthritis in the affected knees [27]. A study conducted at a Level I trauma center [30] reported degenerative meniscal tears in 2 patients (0.09%), while posttraumatic osteoarthritis was observed in 13 (0.6%) patients, 4 of whom required TKA. The same study reported a peroneal nerve entrapment syndrome in one patient and an extensor mechanism deficit in another.

## Discussion

**Fig. 2:**
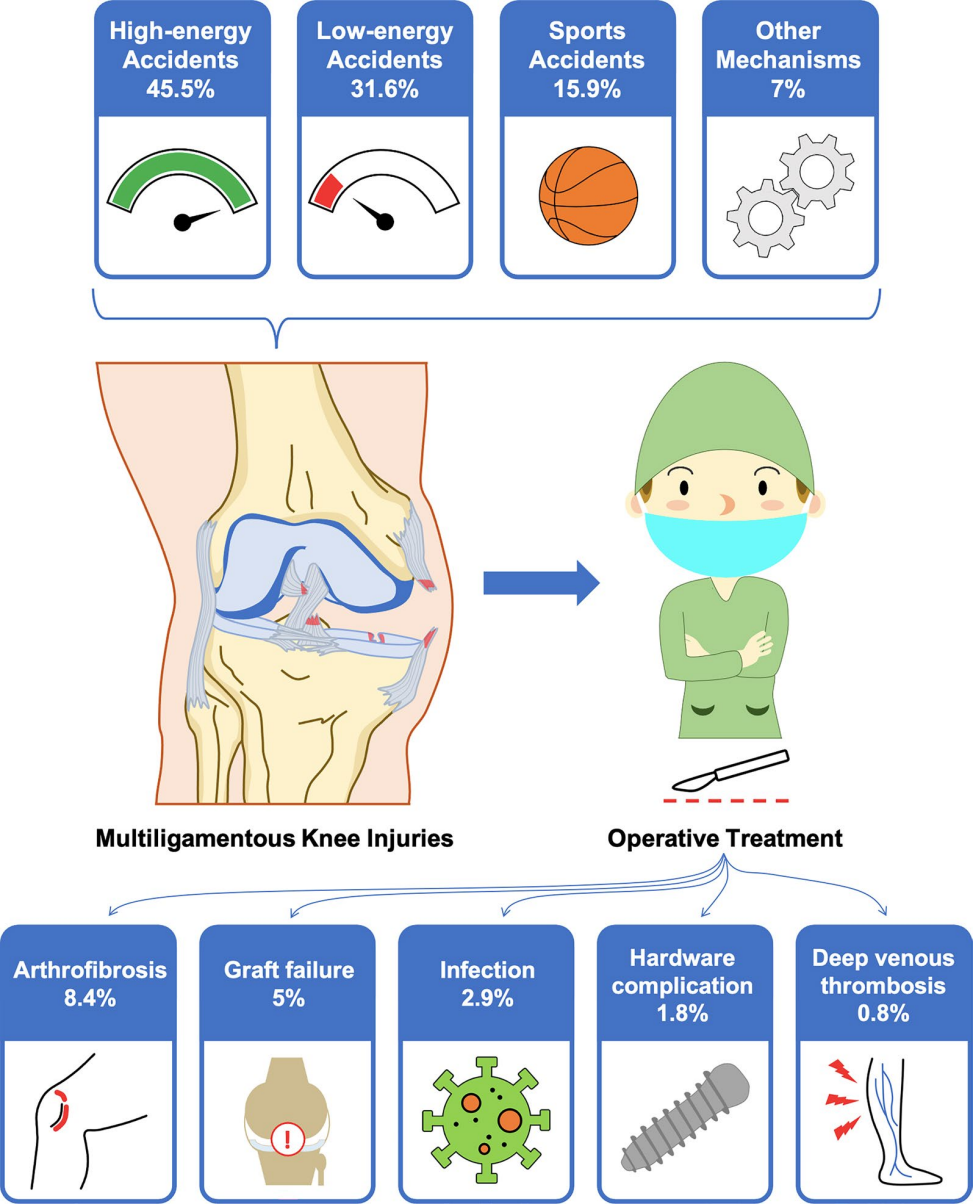
Illustration shows the possible causes of the multiligamentous knee injuries, the need for operative treatment, and the postoperative complications

This systematic review consolidates current evidence on postoperative complications following MLKI surgery, a topic with limited high-quality data and fragmented reporting in existing literature. (Fig. 2). The results show a 19.2% complication rate, and if authors exclude the hardware hassle as a complication, the rate drops to 17.5%. Arthrofibrosis was the most frequently reported complication, with 240 cases requiring surgical management, representing an incidence of 8.4%. Graft failure was observed in 143 cases (5%). Infection was the third most common complication, occurring in 82 cases (2.9%). This review provides clinically relevant insights by identifying the most frequent postoperative complications following MLKI surgery on the basis of a large, diverse sample across multiple studies and surgical approaches. This complication rate is consistent with previous findings, which reported that around 20–30% of patients experienced at least one postoperative complication following MLKI surgery [8, 49].

Arthrofibrosis, a leading cause of stiffness, is a well-recognized and common complication following surgery for MLKIs [50]. This condition is most frequently associated with the severity of the injury and the timing of the initial surgical procedure. Early surgical intervention—specifically within 3 weeks of the injury—is a known risk factor for postoperative stiffness [6, 16, 51]. A standardized definition of stiffness was absent across the studies owing to a lack of consensus on the diagnostic criteria [48, 52]. Early surgical intervention for MLKI improves function and stability while reducing the risk of further articular cartilage damage; however, it also elevates the likelihood of developing stiffness [53]. While arthrofibrosis remains the most usual complication following MLKI surgery, this review found that the incidence of patients requiring reoperation for stiffness is relatively low, at 8.4%. This aligns with findings from another study [51], which reported an arthrofibrosis rate of 11.2% in MLKI cases, demonstrating consistent trends across literature. Similarly, a systematic review [55] analyzing 36 studies with 4159 patients undergoing MLKI surgery identified a postoperative stiffness rate of 9.8%. Notably, this review [55] highlighted that patient with injuries involving only two ligaments had a significantly lower risk of developing postoperative stiffness compared with those with three or more injured ligaments. These findings underscore the multifactorial nature of stiffness risk in MLKI cases and the importance of adjusted surgical and rehabilitation approaches.

**Fig. 3:**
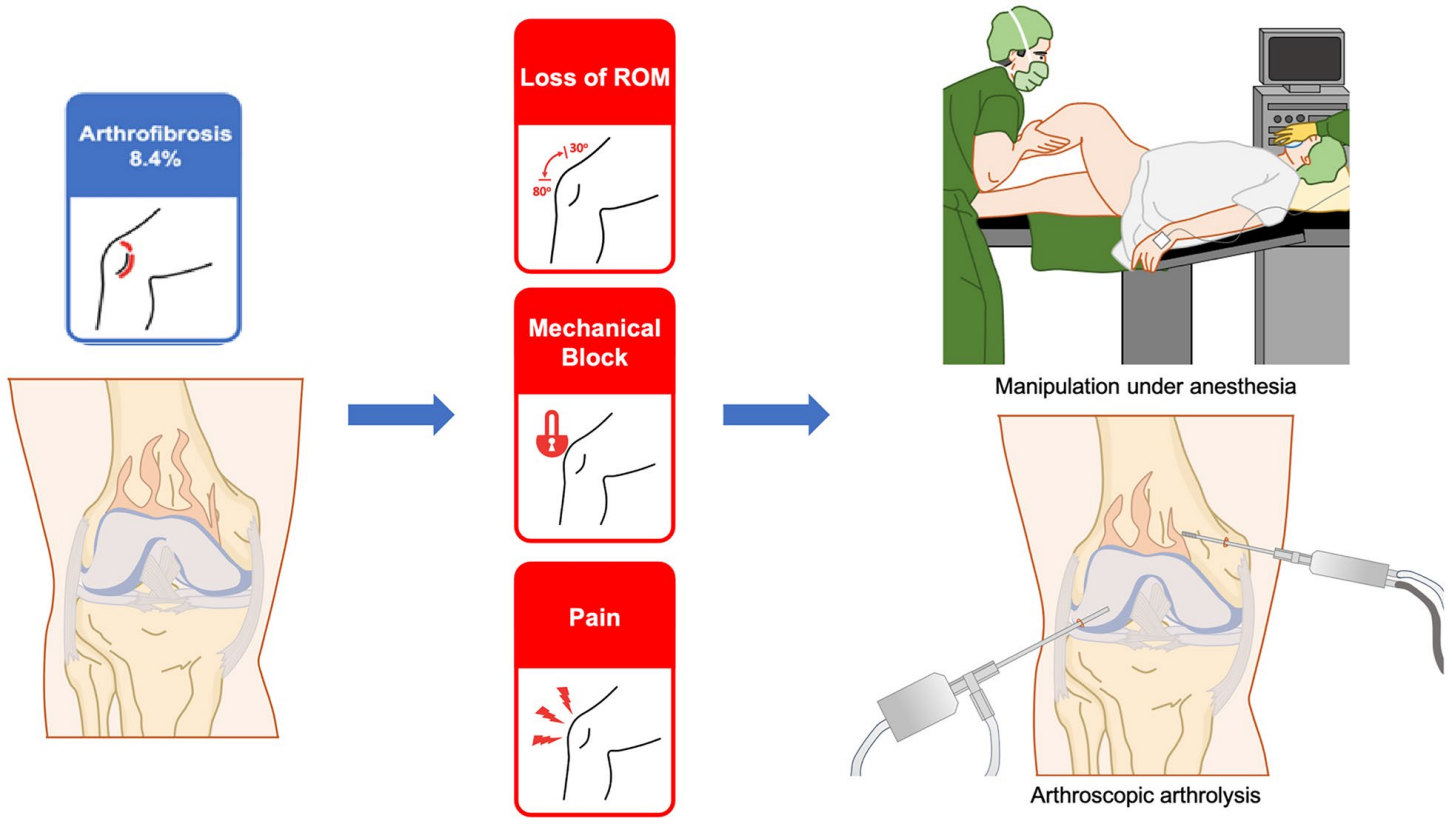
Illustration shows the management of arthrofibrosis, which frequently manifests as stiffness, with manipulations under anesthesia and arthroscopic arthrolysis

Isolated MUA was performed in nine of the studies reviewed, while arthroscopic arthrolysis was utilized in six studies (Fig. 3). Pujol et al. [56] noted that MUA is rarely recommended owing to its significant risks, including fracture, fixation failure, and cartilage damage, although gentle manipulation may be considered within the first 3 months under specific conditions. This finding contrasts with the continued reliance on MUA observed in some of the present studies analyzed, but there was an ongoing shift toward more precise and controlled interventions, such as arthroscopic arthrolysis. Arthroscopic arthrolysis was also frequently employed in the reviewed studies, further solidifying its position as a standard technique for managing stiffness. It is of note that a recent study demonstrated that patients, whether undergoing arthroscopic lysis of arthrofibrosis or not, performed equally well in terms of ROM and clinical scores at a final follow-up of up to 2 years [57]. Moreover, as Pujol et al. emphasized [56], combining arthroscopic and open techniques within a well-structured surgical plan can effectively address various components of stiffness in a single operation. However, the success of these procedures depends critically on closely monitored postoperative care and a supervised rehabilitation program.

Experts generally agree that early operative intervention for MLKI is defined as occurring within 21 days of injury, with delayed intervention taking place beyond this timeframe (level of agreement 76.3% [58]), aligning with most published definitions [7]. This study found that 45.8% of patients underwent delayed reconstruction, while 54.2% underwent early intervention within 21 days of injury. These findings align with literature as there are no consensus on the specific recommendation that early surgery (within 21 days) should always be performed whenever possible [59]. However, as noted by experts, the timing of surgery should ultimately be tailored to individual factors, including the severity and pattern of the MLKI, associated neurovascular injuries, and patient-specific considerations [59]. It is of paramount importance to emphasize that, while acute surgery is strongly associated with ROM deficits, staged procedures may lead to better subjective outcomes and fewer range-of-motion limitations [6, 60]. However, the balance between the advantages of early intervention and the heightened risk of complications, such as stiffness, underscores the need for a patient-centered approach to surgical decision-making.

ROM may be significantly impacted in cases requiring meniscal repair during MLKI treatment. The need for meniscal repair or any form of meniscal intervention has been shown to result in notable decreases in ROM [30]. Additionally, outcomes related to ROM were often worse when injuries involving meniscal tears or the need for meniscal repair accounted [30]. Nevertheless, a study that used partial meniscectomy and meniscus repair found no cases of knee stiffness [23]. Furthermore, two works [19, 25] reported no significant difference in stiffness between patients with MLKI wounds undergoing concurrent meniscal surgery. Meniscal damage may extend rehabilitation timelines, increasing joint immobility and risk of arthrofibrosis. These contrasting findings highlight the need for further research to better understand the impact of meniscal repair on ROM and stiffness in MLKI cases.

On the basis of the review’s data, stiffness rates appear to vary between concomitant medial or lateral collateral ligaments’ injuries, although many studies do not differentiate stiffness outcomes by collateral injury type. In studies where distinctions were made, Ibrahim et al.[15] reported 20% stiffness in 20 lateral injuries, while Freychet et al. [31] observed 7.5% stiffness in 40 lateral injuries. Conversely, medial injuries demonstrated rates such as 7.7% stiffness in 13 cases, as reported by Hongwu et al. [35], and 11.1% stiffness, involving 11 medial injuries, as noted by Goyal et al. [21]. Notably, Tardy et al. [19] differentiated stiffness rates, identifying 18% stiffness across 19 medial and 20 lateral injuries, with management strategies including arthroscopic arthrolysis and mobilization under anesthesia for both group injuries. In several studies, no distinction without further differentiation was made between medial and lateral injuries regarding stiffness outcomes [16, 18, 22, 24, 26, 32, 34, 36, 38]. These results are further supported by Hanley et al. [61], who observed that stiffness was associated with cases involving three or more ligaments requiring operative intervention, though no significant differences were observed on the basis of the specific collateral ligament type involved. These findings suggest that while there may be differences in stiffness rates based on injury location, the current evidence is insufficient to draw definitive conclusions owing to the lack of consistent differentiation in many studies.

Graft failure continues to be a significant complication following MLKI surgery, although it appears less frequently in obese patients, potentially owing to decreased activity levels or biomechanical factors that reduce stress on the grafts [33]. In this review, graft failure was observed in 143 cases, representing 5% of the total sample, but the term was not clearly defined. This aligns closely with the findings of a retrospective case–control study, which reported a comparable graft failure rate of 5.6% for patients undergoing revision ligament surgery [49]. These consistent rates underscore the importance of understanding patient-specific risk factors and surgical techniques to minimize graft failure [62]. Strategies such as careful patient selection, optimizing surgical timing, and addressing modifiable risk factors such as body weight [33], smoking, KD III injuries, and staged reconstruction [49] procedures may help reduce the likelihood of graft failure, thereby improving long-term outcomes in patients with MLKI.

Postoperative infection and wound complications are a persistent concern following surgical treatment of complex MLKIs, especially in cases involving high-energy trauma or injuries characterized by significant initial swelling [63]. Infection emerged as the third most common complication in the present systematic review, with 82 reported cases, representing an incidence of 2.9%. Of these, treatment details were provided for 24 cases: 15 underwent a combination of antibiotics and surgical debridement, while 9 were managed with antibiotics alone. This finding aligns with other studies reporting infection rates of up to 6.5%, requiring irrigation and debridement, and incidence rates ranging from 0.30% to 12.5% in open reconstructions [49, 64]. Factors such as advanced age, compromised general health, poor skin condition, steroid use, extended tourniquet time, and prior knee surgeries have been identified as contributors to infection risk [54, 64]. Prophylactic antibiotic therapy administered for 24 h pre- and post-surgery (or until drain removal) has been shown to significantly reduce infection rates [63]. Additionally, careful wound closure techniques are essential, as excessive traction during closure can lead to blistering and increased infection risk [64]. These findings underscore the importance of meticulous surgical techniques, perioperative care, and patient-specific risk assessment to minimize infection-related complications in ligamentous knee surgeries[65].

## Limitations

This review has several limitations that should be acknowledged. First, the generally moderate quality of the included studies, as indicated by the MINORS assessment, suggests that the findings of this systematic review should be interpreted with caution. Second, the majority of the included studies were retrospective, which may introduce publication bias and limit the ability to capture all relevant data. Studies were heterogeneous, with generally low-quality evidence and relatively short follow-up (minimum 12 months), limiting long-term conclusions. Additionally, the lack of direct comparative studies and long-term follow-up may obscure important outcomes, such as graft failure or the durability of surgical reconstruction or repair. The heterogeneity among the included studies, particularly in patient selection criteria and injury patterns, further complicates the ability to draw robust conclusions. Variability in surgical techniques, timing of intervention, and rehabilitation protocols also make direct comparisons challenging. Furthermore, authors were unable to conduct subgroup analyses to assess the impact of specific variables, such as the type of ligament repair versus reconstruction or the influence of injury severity on complications such as stiffness or infection. The variability in surgical techniques, rehabilitation protocols, follow-up duration, and reporting methods for complications made it challenging to conduct a meaningful and reliable subgroup analysis. Moreover, only studies published in English were included, potentially excluding relevant data from non-English sources. Despite these limitations, this review offers valuable insights into the complications of MLKIs and specifically to stiffness while adhering to PRISMA guidelines.

## Conclusions

MLKIs present significant challenges owing to their complexity and high complication rates. This review provides valuable insights into the management and rates of arthrofibrosis, infection, and graft failure, reinforcing the need for individualized, evidence-based approaches. Future studies should focus on prospective designs, long-term follow-up, and standardized protocols to improve outcomes in this challenging patient population.

## Data Availability

The datasets used and/or analyzed during the current study are available from the corresponding author on reasonable request.
